# Knowledge, Attitudes, and Practices Regarding Antibiotic Use Among the General Population in Kuwait: A Cross-Sectional Study

**DOI:** 10.7759/cureus.100890

**Published:** 2026-01-06

**Authors:** Moosa N Mohiyaldeen, Khaled J Alsamhan, Abdullah B AlRukaibi, Reem S AlSawwagh, Maram W AlZanki, Munirah Ebrahim, Zainab Abulhasan, Zainab Al Mousa, Abdullah Almajran

**Affiliations:** 1 Faculty of Medicine, Kuwait University, Kuwait City, KWT; 2 Community Medicine, Kuwait University, Kuwait City, KWT

**Keywords:** antibiotic, attitudes, knowledge, prevention, resistance

## Abstract

Background

Antibiotic resistance is a growing global public health threat, largely driven by inappropriate use and misuse of antibiotics in the community. Understanding public knowledge, attitudes, and practices regarding antibiotic use is essential for designing effective interventions.

Objectives

The objective of this study was to assess the general population of Kuwait’s knowledge, attitudes, and proper use of antibiotics.

Methods

This was a cross-sectional study that used a validated questionnaire. Participants had to be 18 years and above, residing in Kuwait, and of any nationality. The data collection period was from the 27th of December 2023 to the 7th of January 2024. The sample size of this study was 743 participants. All statistical analyses were conducted using IBM Corp. Released 2023. IBM SPSS Statistics for Windows, Version 29. Armonk, NY: IBM Corp.

Results

Almost all participants (96.1%) displayed a low to moderate level of knowledge regarding antibiotics. Attitudes towards antibiotics were positive in 86% of the study population. Approximately 53.16% of individuals enrolled in the study do not use antibiotics properly.

Conclusion

The overwhelming majority of participants displayed low to moderate knowledge about antibiotics, which is consistent with data from the local region. However, attitudes towards antibiotics were resoundingly positive amongst participants. On the contrary, more than half of the participants did not exhibit proper use of antibiotics. The findings of this study will guide policymakers in Kuwait to implement tailored interventions focusing on increasing knowledge and reinforce medical practitioners to explain the proper use of antibiotics to patients.

## Introduction

The surge in antibiotic resistance poses a substantial threat to global health [1]. Antibiotic resistance occurs when bacteria evolve to withstand medications designed to eliminate them and is driven primarily by the misuse and overuse of antibiotics in healthcare, agriculture, and livestock. If current trends persist, antibiotic resistance is estimated to cause 10 million deaths worldwide [2]. Inappropriate antibiotic use, including incomplete treatment courses, accelerates the emergence of resistant strains, leading to infections that are increasingly difficult to treat and associated with higher morbidity and mortality [3]. This threat is further intensified by the rise of multidrug-resistant bacteria, which limits available treatment options [4].

In Kuwait, antibiotic resistance has increased markedly, with studies reporting a 17% growth rate in resistance between 1999 and 2003 [5], a rise partly attributed to inappropriate antibiotic use. A 2022 study assessing knowledge and attitudes toward antibiotic use across 11 Arab countries found that misconceptions regarding antibiotic effectiveness and indications remained prevalent, affecting 73% of respondents [6]. In Kuwait, a recent cross-sectional survey reported that 36% of individuals prescribed antibiotics within the past 12 months did not complete their treatment course [1]. While previous studies have examined antibiotic knowledge and practices in Kuwait, this study aimed to further assess antibiotic use among the general population and explore the reasons underlying misuse. The World Health Organization has identified antibiotic resistance as a serious public health concern in Kuwait. Despite numerous international studies on antibiotic use [7,8], research in Kuwait remains limited, with no nationwide surveillance system and available data largely derived from individual hospital-based studies [9]. Therefore, the objectives of this study were to assess the population’s knowledge, attitudes, and proper use of antibiotics.

## Materials and methods

Study design and population

This was an observational cross-sectional study conducted to assess knowledge, attitudes, and use of antibiotics in the general population in Kuwait. The study was performed through distributing an anonymous questionnaire with 39 questions that was adapted from validated surveys, both in English and Arabic, that were previously used in Kuwait and Lebanon. Both of these studies underwent pilot testing and were validated. Those questionnaires were Knowledge, Attitude, and Practice Towards Antibiotic Use Among the Public [1] and Knowledge, Attitude, and Practice on Antibiotic Use [10]. This study was approved by the Ministry of Health’s Standing Committee for Coordination of Health and Medical Research, and the ethical approval number is 542. Informed consent was obtained from all participants and completed electronically. The data collection period was from the 27th of December 2023 to the 7th of January 2024. The data collection period was intentionally short due to the use of an electronic questionnaire distributed in high-traffic community settings, which enabled rapid recruitment. The short study duration minimized temporal variation in antibiotic-related behaviors and attitudes while allowing the study to exceed the required sample size within a limited timeframe.

Sampling and inclusion criteria

Inclusion criteria for the study were individuals living in Kuwait, both Kuwaiti and non-Kuwaiti, and over the age of 18 years. The target sample size was approximately 500 participants. The minimum required sample size was calculated as 384 participants using a 95% confidence interval based on the population of Kuwait. To improve statistical power, allow for subgroup analyses, and account for incomplete or invalid responses, a higher target sample size of approximately 500 participants was set. Ultimately, 743 complete responses were collected during the study period, exceeding the minimum required sample size.

Convenience sampling was used for participant selection, which is a non-probability sampling technique where participants are selected based on their availability and proximity to the researchers, as it is cost-effective, feasible, time-efficient, and useful for exploratory research. Data collection was done in several community settings, aiming to get a broader understanding of the topic across different community contexts, including the Ministry of Justice, Palace of Justice, Ministry of Education, Ministry of Social Affairs, Ministries complex, Public Authority of Civil Information, and Directorate General of Civil Aviation. Data collection was also done in Al-Assima Mall. We requested individuals aged 18 years and older to fill out the questionnaire upon scanning the quick response code. Members of the research team were available to answer participants’ questions as they were completing the questionnaire. 

Study questionnaire

The first section of the questionnaire consisted of 12 demographic questions, including age, gender, marital status, nationality, level of education, employment status, monthly personal income, governorate, existing medical conditions, and being a health care practitioner or having a family member who is a health care practitioner. The second section consisted of 13 statements and assessed the population’s knowledge of antibiotics, their side effects, and knowledge about antibiotic resistance using a five-point Likert scale (1 = Strongly Disagree; 5 = Strongly Agree). Those statements were adapted and tailored from the validated survey previously used in Kuwait [1].

The knowledge score was categorized as low (0-6 points), moderate (7-10 points), and high (11-13 points). A point was given for answering ‘Agree’ or ‘Strongly Agree’ to correct knowledge statements, and a point was given for answering ‘Disagree’ or ‘Strongly Disagree’ to incorrect knowledge statements. Neutral answers were scored as 0. Total scores could range from 0 to 13 points.

The third section included six statements assessing respondents’ attitudes that were adapted from the validated survey previously used in Kuwait [1]. Respondents were required to answer with yes or no. All the statements listed in this section were negative. If participants answered ‘yes’, they got 1 point. Participants were deemed to have positive attitudes if their total score was 0-3 points, or negative attitudes if the total score was 4-6 points.

Finally, the fourth section consisted of nine questions to assess the population’s use of antibiotics over the past year, which were adapted from the survey previously used in Lebanon [10]. To be categorized as using antibiotics correctly, answers are supposed to be as follows: for question 2, as being prescribed antibiotics by the doctor; for question 3, they had to answer with a ‘yes’; correct answers for question 4 were either ‘I disposed of them’ or ‘I don’t have any remaining antibiotics,’ as well as ‘not applicable’ to both questions 5 and 6.

Based on their responses, participants were categorized as either having one or more incidences of improper antibiotic use or having no incidences of improper use.

Statistical analysis

Descriptive analysis was used to obtain frequencies and estimate proportions. Associations between knowledge scores and different variables were evaluated using parametric tests; the t-test was used for variables with two categories, and one-way analysis of variance (ANOVA) was applied for variables with three or more categories. To reduce type I error, the Bonferroni correction method was utilized in coherence with the ANOVA test. Furthermore, associations between correct use and both knowledge and attitudes within different variables were computed using Pearson Chi-Square tests, and trends were assessed using Linear-by-Linear association with regard to ordinal variables. Additionally, a logistic regression model was generated to assess the odds of proper antibiotic use in relation to different variables. The logistic regression model was tested for significance using the Omnibus test, and the Hosmer and Lemeshow test was used to assess whether the model fit the data well. P-values < 0.05 were considered statistically significant.

## Results

Descriptive analysis

Table 1 represents the demographics of the participants in this study. A total of 743 participants completed the questionnaire. The study population included adults across all age groups, with a predominance of younger participants. Most respondents were female and of Kuwaiti nationality. The majority had a bachelor’s degree or higher, and approximately one-fifth reported having a chronic medical condition. Most participants were not healthcare practitioners, although many reported having a family member or friend working in healthcare.

**Table 1:** Demographics *Includes diabetes mellitus, heart disease, asthma, COPD, high blood pressure, and high cholesterol

Characteristic	n (%)
Age (years)	
18-25	247 (33.3)
26-36	233 (31.4)
37-50	188 (25.3)
>50	75 (10.1)
Gender	
Male	219 (29.5)
Female	524 (70.5)
Marital status	
Single	338 (45.5)
Married	374 (50.3)
Divorced/Widowed	31 (4.2)
Do you have children?	
Yes	372 (50.1)
No	371 (49.9)
Nationality	
Kuwaiti	634 (85.3)
Non-Kuwaiti	109 (14.7)
Level of Education	
High school or less	104 (14.0)
Diploma	115 (15.5)
Bachelor’s degree	463 (62.3)
Graduate student	61 (8.2)
Employment status	
Student	172 (23.1)
Employed	479 (64.5)
Unemployed	47 (6.3)
Retired	45 (6.1)
Monthly personal income	
Less than 500 KD	153 (20.6)
500-999 KD	176 (23.7)
1000-1500 KD	252 (33.9)
More than 1500 KD	162 (21.8)
In which governate do you live?	
Al-Ahmadi	68 (9.2)
Al-Assima	167 (22.5)
Hawalli	171 (23.0)
Al-Jahra	73 (9.8)
Al-Farwaniya	170 (22.9)
Mubarak Al-Kabeer	94 (12.7)
Do you have any chronic diseases? *	
Yes	168 (22.6)
No	575 (77.4)
Are you a healthcare practitioner (Medical doctor, dentist, pharmacist, nurse)?	
Yes	119 (16.0)
No	624 (84.0)
Do you have a family member or a friend who is a healthcare practitioner (Medical doctor, dentist, pharmacist, nurse)	
Yes	514 (69.2)
No	229 (30.8)

Assessing the general population’s knowledge of antibiotics

Overall, participants demonstrated predominantly low to moderate knowledge regarding antibiotics (Table 2). While most respondents correctly identified that antibiotics are effective against bacteria and that different antibiotics are required for different diseases, misconceptions were common. A substantial proportion of participants believed that antibiotics accelerate recovery from coughs and colds and that antibiotics are effective against viral infections. Knowledge gaps were also observed regarding antibiotic resistance, with many participants either uncertain or unaware that resistance is a global problem. Notably, the majority incorrectly believed that humans themselves can become resistant to antibiotics.

**Table 2:** Knowledge of antibiotics Scoring and questions were derived from the KAP questionnaire [1]. ** 'Agree’ or ‘strongly agree’ to correct statements and ‘disagree’ or ‘strongly disagree’ to incorrect statements = 1 point. All other answers = 0 points.

Statements on Knowledge	Strongly Disagree n (%)	Disagree n (%)	Neutral n (%)	Agree n (%)	Strongly Agree n (%)	Correct or incorrect statements**
Different antibiotics are needed to cure different diseases	27 (3.6)	37 (5.0)	128 (17.2)	417 (56.1)	134 (18.0)	Correct
Antibiotics are effective against bacteria	17 (2.3)	37 (5.0)	162 (21.8)	400 (53.8)	127 (17.1)	Correct
Antibiotics can kill the bacteria that normally on the skin and the gut	17 (2.3)	48 (6.5)	231 (31.1)	370 (49.8)	77 (10.4)	Correct
Antibiotics speed up the recovery for most coughs and colds	45 (6.1)	106 (14.3)	130 (17.5)	344 (46.3)	118 (15.9)	Incorrect
Antibiotics work on most cough and colds	49 (6.6)	144 (19.4)	156 (21.0)	311 (41.9)	83 (11.2)	Incorrect
Antibiotics are effective against viruses	108 (14.5)	141 (19.0)	174 (23.4)	252 (33.9)	68 (9.2)	Incorrect
If you get side effects during a course of antibiotics treatment, you should stop taking them as soon as possible	27 (3.6)	53 (7.1)	103 (13.9)	328 (44.1)	232 (31.2)	Correct
If you get some kind of skin reaction when using antibiotics, you should not use the same antibiotic again	30 (4.0)	42 (5.7)	77 (10.4)	322 (43.3)	272 (36.6)	Correct
Antibiotics can cause an imbalance of the body’s own bacterial flora	10 (1.3)	46 (6.2)	254 (34.2)	305 (41.0)	128 (17.2)	Correct
The unnecessary use of antibiotics can increase the resistance of bacteria to them	19 (2.6)	50 (6.7)	166 (22.3)	292 (39.3)	216 (29.1)	Correct
Resistance to antibiotics is a worldwide problem	7 (0.9)	46 (6.2)	270 (36.3)	297 (40.0)	123 (16.6)	Correct
The use of antibiotics among animals can reduce the effect of antibiotics among humans	39 (5.2)	144 (19.4)	391 (52.6)	136 (18.3)	33 (4.4)	Correct
Humans can be resistant to antibiotics	14 (1.9)	41 (5.5)	202 (27.2)	359 (48.3)	127 (17.1)	Incorrect

Table 3 represents the mean differences in knowledge scores. The mean score of knowledge of antibiotics was significantly lower in Kuwaiti participants compared to non-Kuwaitis. Furthermore, participants with graduate degrees scored significantly higher in comparison to participants with lower educational degrees, which was assessed using a post-hoc analysis. In addition, those with a monthly personal income of more than 1500 KD scored significantly higher than those in lower income categories. Participants who were healthcare practitioners also scored significantly higher compared to those who were not. No significant differences in knowledge scores were observed across age groups, gender, marital status, governorate of residence, or presence of chronic disease.

**Table 3:** Mean difference in knowledge of antibiotic scores across different demographic characteristics *Mean percentage of correct answers on the antibiotic knowledge questionnaire (highest at 92% and lowest at 0%) **Denotes significance at p<0.05 ***Diabetes Mellitus, heart disease, asthma, COPD, high blood pressure, high cholesterol. ****Post hoc analysis was used to obtain the p-values.

Characteristics	Mean (±SD) *	p-value
Age		0.198
18 to 25	49.3 (±18.7)	
26 to 36	49.1 (±17.6)	
37 to 50	52.5 (±17.4)	
> 50	50.8 (±16.3)	
Gender		0.361
Male	51.2 (±18.6)	
Female	49.8 (±17.5)	
Marital status		0.965
Single	50.4 (±18.2)	
Married	50.2 (±17.7)	
Divorced/widowed	49.6 (±15.2)	
Do you have any children?		0.608
No	49.9 (±18.1)	
Yes	50.6 (±17.5)	
Nationality		0.003**
Kuwaiti	49.4 (±17.5)	
Non-Kuwaiti	54.9 (±18.7)	
Level of education		0.005**
High school or less	50.5 (±16.9)	
Diploma	47.0 (±15.4)	
Bachelor's degree	50.1 (±18.1)	
Graduate degree	57.1 (±20.0)	
Employment status		0.054
Student	51.3 (±18.8)	
Employed	49.3 (±17.5)	
Unemployed	56.4 (±17.2)	
Retired	49.9 (±17.1)	
Monthly personal income		0.012**
Less than 500 KD	49.5 (±17.9)	
500-999 KD	49.4 (±17.4)	
1000-1500 KD	48.7 (±17.4)	
More than 1500 KD	54.3 (±17.8)	
In which governorate do you live?		0.579
Al-Ahmadi	48.3 (±17.3)	
Al-Assima	50.4 (±16.3)	
Hawalli	51.1 (±19.1)	
Al-Jahra	48.2 (±20.3)	
Al-Farwaniya	51.6 (±17.1)	
Mubarak Al-Kabeer	48.8 (±17.5)	
Do you have any chronic diseases? ***		0.516
No	50.5 (±18.0)	
Yes	49.4 (±17.3)	
Are you a healthcare practitioner (Medical doctor, dentist, pharmacist, nurse)?		<0.001**
No	48.7 (±17.5)	
Yes	58.1 (±17.6)	
Do you have a family member or a friend who is a healthcare practitioner (Medical doctor, dentist, pharmacist, nurse)?		0.891
No	50.4 (±17.0)	
Yes	50.2 (±18.2)	

Assessing the general population’s attitudes towards antibiotics

Table 4 displays that most participants exhibited positive attitudes toward antibiotic use. The majority disagreed with obtaining antibiotics from friends, relatives, or pharmacies without a prescription. However, a considerable proportion reported attitudes that may contribute to misuse, including keeping antibiotics for future use and preferring antibiotics for prolonged cough or sore throat. Overall, the majority of participants were categorized as having positive attitudes toward antibiotic use.

**Table 4:** Attitude towards antibiotics

Negative Attitude Statements	Yes n (%)	No n (%)
It is good to be able to get antibiotics from relatives or friends without having to see a medical doctor	47 (6.3)	696 (93.7)
I prefer to be able to get antibiotics from the pharmacy without a prescription	90 (12.1)	653 (87.9)
I prefer to keep antibiotics at home in case there may be a need for them later	257 (34.6)	486 (65.4)
If I feel better after a few days, I sometimes stop taking my antibiotics before completing the course of treatment	238 (32.0)	505 (68.0)
I prefer to use an antibiotic if I have a cough for more than a week	337 (45.4)	406 (54.6)
When I have a sore throat, I prefer to use an antibiotic	340 (45.8)	403 (54.2)

Assessing the general population's proper use of antibiotics.

Table 5 shows that antibiotic use was common among participants, with most reporting antibiotic use within the past year, typically limited to a few courses. Antibiotics were predominantly prescribed or recommended by physicians, while non-medical sources such as family members, friends, pharmacists, or leftover medications at home were less frequently reported. Most participants indicated that they completed the prescribed course of treatment; however, a considerable proportion reported not doing so. Regarding leftover antibiotics, disposal was the most commonly reported practice, although many participants reported keeping unused antibiotics for future use. Self-adjustment of antibiotic dosage was relatively uncommon, with most participants indicating that dose increases or reductions were not applicable. Among those who altered their dosage, reasons included forgetting doses, concerns about side effects, feeling better, or perceiving insufficient improvement. Around half of the participants reported that they were either never instructed or only sometimes received instructions on proper antibiotic usage from their physician.

**Table 5:** Use of antibiotics

Questions on antibiotic use	n (%)
Over the past year, how many times did you use antibiotics?	
0 times	175 (23.6)
1-3 times	462 (62.2)
4-6 times	59 (7.9)
More than 6 times	47 (6.3)
Who prescribed or recommended the antibiotics?	
The doctor	659 (88.7)
Friends	15 (2.0)
Family	20 (2.7)
Pharmacist	28 (3.8)
I had them at home	21 (2.8)
The last time you had to take antibiotics, did you complete the course of treatment?	
Yes	524 (70.5)
No	219 (29.5)
What did you do with the antibiotics that were left unused?	
I kept them to be used the next time I am sick	167 (22.5)
I disposed them	385 (51.8)
I gave them to someone	18 (2.4)
I don’t have any remaining antibiotics	173 (23.3)
If you have ever increased your dose of antibiotics, why did you increase the dose on your own?	
Not applicable	658 (88.6)
I forgot to take the previous dose	34 (4.6)
I felt very sick	24 (3.2)
I didn’t feel a notable improvement	21 (2.8)
I felt better but wanted to improve even more	6 (8.0)
If you have ever reduced your dose of antibiotics, why did you reduce the dose on your own?	
Not applicable	606 (81.6)
I was worried about the medication’s side effects	16 (2.2)
I was tired and I forgot	41 (5.5)
I felt that I was taking too many medications	10 (1.3)
I was in a hurry and I forgot	12 (1.6)
I don’t like taking medicines at night	5 (0.7)
I was feeling better	53 (7.1)
When you are prescribed antibiotics by your doctor, are you instructed on their proper use?	
Never	56 (7.5)
Sometimes	256 (34.5)
Always	431 (58.0)

Our data showed that around half of the participants displayed at least one behavior reflecting improper use. Table 6 displays the binary logistic regression model for antibiotic use. Individuals over the age of 50 years were three times more likely to properly use antibiotics compared to 18-25-year-olds. In comparison to males, females had 1.52 times higher adjusted odds of proper use. With regard to knowledge of antibiotics, the odds of proper use were three times higher in those with high knowledge scores compared to those with low knowledge scores. Furthermore, individuals with a negative attitude towards antibiotics were significantly less likely to use antibiotics properly than those with positive attitudes. Additionally, individuals who reported always receiving instructions on proper antibiotic use were more than two times more likely to use them properly, compared to those who had never been instructed.

**Table 6:** Logistic regression of different characteristics in relation to proper use of antibiotics *Denotes significance at p<0.05 **Includes diabetes mellitus, heart disease, asthma, COPD, high blood pressure, and high cholesterol ***Adjusted for age, gender, marital status, if they have children, levels of education, employment status, monthly personal income, governorate, having chronic diseases, knowledge category, attitude category, and if they have been instructed on their proper use by their doctor

Factor	Adjusted OR***	Adjusted 95% CI	Adjusted p-value
Age			
18-25 (reference)			0.020*
26-36	0.940	0.519-1.702	0.838
37-50	1.706	0.840-3.463	0.140
>50	2.899	1.128-7.449	0.027*
Gender			
Male (reference)			
Female	1.526	1.022-2.280	0.039*
Marital Status			
Single (reference)			0.683
Married	1.234	0.599-2.543	0.569
Divorced/Widowed	0.891	0.312-2.545	0.829
Do you have any children?			
No (reference)			
Yes	1.387	0.690-2.788	0.359
Nationality			
Kuwaiti (reference)			
Non-Kuwaiti			
Level of education			
High school or less (reference)			0.263
Diploma	1.005	0.500-2.020	0.989
Bachelor’s degree	1.307	0.750-2.279	0.345
Graduate	2.002	0.899-4.457	0.089
Employment Status			
Student (reference)			0.172
Employed	0.562	0.287-1.098	0.092
Unemployed	0.918	0.372-2.265	0.852
Retired	1.023	0.328-3.188	0.969
Monthly Income			
Less than 500 KD (reference)			0.569
500-999 KD	1.435	0.829-2.483	0.197
1000-1500 KD	1.427	0.799-2.550	0.229
More than 1500 KD	1.482	0.748-2.935	0.260
In which Governorate do you live?			
Al-Ahmadi (reference)			0.206
Al-Assima	1.597	0.820-3.111	0.169
Hawalli	1.938	0.993-3.779	0.052
Al-Jahra	0.929	0.417-2.070	0.857
Al-Farwaniya	1.644	0.843-3.208	0.145
Mubarak Al-Kabeer	1.350	0.649-2.809	0.422
Do you have any chronic diseases? **			
No (reference)			
Yes	1.386	0.902-2.129	0.137
Are you a healthcare practitioner?			
No (reference)			
Yes			
Do you have a family member or a friend who is a healthcare practitioner?			
No (reference)			
Yes			
Knowledge Category			
Low (reference)			0.033*
Moderate	1.337	0.954-1.873	0.092
High	3.005	1.167-7.733	0.023*
Attitude Category			
Positive (reference)			
Negative	0.107	0.052-0.217	<0.001*
When you are prescribed antibiotics by your doctor, are you instructed on their proper use?			
Never (reference)			<0.001*
Sometimes	1.292	0.628-2.658	0.487
Always	2.440	1.205-4.942	0.013*

## Discussion

This study assessed knowledge, attitudes, and practices regarding antibiotic use among the general population in Kuwait. The findings indicate that while attitudes toward antibiotic use were largely positive, knowledge levels remained predominantly low to moderate, and improper antibiotic use was common. Importantly, higher knowledge levels, positive attitudes, older age, female gender, and receiving instructions from healthcare providers were independently associated with proper antibiotic use.

With regard to knowledge, participants showed a reasonable understanding of some fundamental antibiotic concepts, such as the need for different antibiotics to treat different diseases and the role of antibiotics in treating bacterial infections. These findings are broadly consistent with those reported in a previous Kuwaiti study by Awad et al., although our participants demonstrated slightly better performance on certain knowledge items [1]. This improvement may reflect increased public awareness over the past decade, potentially resulting from antibiotic awareness campaigns and improved access to health information [11]. Nevertheless, persistent misconceptions were observed, particularly regarding the use of antibiotics for viral infections such as coughs and colds. Similar gaps in knowledge have been reported in regional and international studies, including those conducted in Lebanon and Indonesia, while studies from countries such as Sweden have demonstrated lower levels of such misconceptions [12]. The overall level of antibiotic knowledge observed in this study is comparable to findings from neighboring countries [1,12], with marginally higher knowledge possibly attributable to the relatively high educational attainment of the study population and improved patient-provider communication. 

In terms of attitudes and practices, most participants reported obtaining antibiotics through physicians rather than non-medical sources, reflecting an improvement compared with earlier local and regional studies that reported higher levels of non-prescription antibiotic use. However, inappropriate practices persist. A notable proportion of participants reported not completing prescribed antibiotic courses, keeping leftover antibiotics for future use, or sharing antibiotics with others. These behaviors have also been documented in previous studies conducted in Kuwait, Jordan, and other countries, indicating that improper antibiotic use remains a widespread public health concern [13]. Compared with studies from Korea and other settings, the proportion of participants failing to complete antibiotic courses in this study was lower but still substantial [14]. This may be due to the participants in our study having a higher level of education. In addition, about half of the participants prefer to use antibiotics for prolonged cough, even though there is evidence to support the prescription of antibiotics for such cases [15].

Improper handling of leftover antibiotics, including storing or sharing them, poses risks not only to individual health but also to the broader environment, as inappropriate disposal may contribute to the development and spread of antibiotic-resistant bacteria [14]. Although most participants reported not altering their prescribed antibiotic dose, those who did so commonly increased the dose to hasten recovery or reduced it after feeling better. Such practices are concerning, as increasing doses may lead to adverse drug effects [16], while reducing doses or failing to complete treatment can promote bacterial resistance and relapse. These findings are consistent with previous research highlighting treatment non-adherence as a major contributor to antimicrobial resistance in Kuwait and globally [5,17-19].

Importantly, this study found that participants who reported receiving clear instructions from healthcare providers were more likely to use antibiotics appropriately. This underscores the critical role of physicians and other healthcare professionals in promoting correct antibiotic use through effective counseling and communication. Similar conclusions have been reported by international health authorities, including the Centers for Disease Control and Prevention, which emphasize that a substantial proportion of outpatient antibiotic use remains inappropriate [20]. Collectively, these findings highlight the need for sustained public education, strengthened antimicrobial stewardship programs, and policies aimed at improving patient education and regulating antibiotic access to mitigate the growing threat of antibiotic resistance. Addressing knowledge discrepancies amongst various demographic categories through targeted educational interventions can educate individuals to make informed choices about antibiotic usage, encouraging responsible practices and minimizing unnecessary demand for these medications. Furthermore, comprehending public attitudes towards antibiotics enables healthcare professionals to tailor their communication strategies, promoting a collaborative approach to antibiotic utilization. In addition, the pivotal findings of this study will guide policymakers in Kuwait to formulate and implement future interventions aiming to enhance the proper use of antibiotics. 

Limitations

Despite the large sample size and use of validated questionnaires that strengthen the reliability of the findings, this study has several limitations. The use of convenience sampling limits generalizability, and the overrepresentation of females and highly educated individuals may introduce selection bias. Additionally, recall bias is possible due to self-reported antibiotic use. Recall bias is also possible, as respondents were asked about their use of antibiotics over the past year.

Implications

Assessing the knowledge and use of antibiotics within the general population carries substantial implications for public health. The improper use and excessive consumption of antibiotics contribute significantly to the rise of antibiotic-resistant bacteria, which poses a serious threat to the efficacy of these crucial medications. A comprehensive assessment of these factors can provide insights into the knowledge of antibiotic principles and awareness regarding antibiotic resistance. Addressing knowledge discrepancies amongst various demographic categories through targeted educational interventions can educate individuals to make informed choices about antibiotic usage, encouraging responsible practices and minimizing unnecessary demand for these medications. Furthermore, comprehending public attitudes towards antibiotics enables healthcare professionals to tailor their communication strategies, promoting a collaborative approach to antibiotic utilization. In addition, the pivotal findings of this study will guide policymakers in Kuwait to formulate and implement future interventions aiming to enhance the proper use of antibiotics.

## Conclusions

This study demonstrates that despite generally positive attitudes toward antibiotics among the population in Kuwait, knowledge gaps and improper antibiotic use remain prevalent. Proper antibiotic use was strongly associated with higher knowledge levels and receiving clear instructions from healthcare providers. These findings underscore the need for targeted public education initiatives and enhanced patient-provider communication to promote responsible antibiotic use and combat antimicrobial resistance.
